# Chronic pancreatitis: Multicentre prospective data collection and analysis by the Hungarian Pancreatic Study Group

**DOI:** 10.1371/journal.pone.0171420

**Published:** 2017-02-16

**Authors:** Ákos Szücs, Tamás Marjai, Andrea Szentesi, Nelli Farkas, Andrea Párniczky, György Nagy, Balázs Kui, Tamás Takács, László Czakó, Zoltán Szepes, Balázs Csaba Németh, Áron Vincze, Gabriella Pár, Imre Szabó, Patrícia Sarlós, Anita Illés, Szilárd Gódi, Ferenc Izbéki, Judit Gervain, Adrienn Halász, Gyula Farkas, László Leindler, Dezső Kelemen, Róbert Papp, Richárd Szmola, Márta Varga, József Hamvas, János Novák, Barnabás Bod, Miklós Sahin-Tóth, Péter Hegyi, on behalf of the Hungarian Pancreatic Study Group

**Affiliations:** 1 First Department of Surgery, Semmelweis University, Budapest, Hungary; 2 Institute for Translational Medicine, University of Pécs, Pécs, Hungary; 3 First Department of Medicine, University of Szeged, Szeged, Hungary; 4 Institute of Bioanalysis, University of Pécs, Pécs, Hungary; 5 Heim Pál Children’s Hospital, Budapest, Hungary; 6 Second Department of Internal Medicine, University of Debrecen, Debrecen, Hungary; 7 Division of Gastroenterology, First Department of Medicine, University of Pécs, Pécs, Hungary; 8 Division of Translational Medicine, First Department of Medicine, University of Pécs, Pécs, Hungary; 9 Szent György University Teaching Hospital of Fejér County, Székesfehérvár, Hungary; 10 Department of Surgery, University of Szeged, Szeged, Hungary; 11 Department of Surgery, University of Pécs, Pécs, Hungary; 12 Department of Interventional Gastroenterology, National Institute of Oncology, Budapest, Hungary; 13 Dr. Réthy Pál Hospital, Békéscsaba, Hungary; 14 Bajcsy-Zsilinszky Hospital, Budapest, Hungary; 15 Pándy Kálmán Hospital of Békés County, Gyula, Hungary; 16 Dr. Bugyi István Hospital, Szentes, Hungary; 17 Center for Exocrine Disorders, Department of Molecular and Cell Biology, Boston University Henry M. Goldman School of Dental Medicine, Boston, Massachusetts, United States of America; 18 Hungarian Academy of Sciences—University of Szeged, Momentum Gastroenterology Multidisciplinary Research Group, Szeged, Hungary; Cedars-Sinai Medical Center, UNITED STATES

## Abstract

**Introduction:**

Chronic pancreatitis is an inflammatory disease associated with structural and functional damage to the pancreas, causing pain, maldigestion and weight loss and thus worsening the quality of life.

**Aims and methods:**

Our aim was to find correlations from a multicentre database representing the epidemiological traits, diagnosis and treatment of the disease in Hungary. The Hungarian Pancreatic Study Group collected data prospectively from 2012 to 2014 on patients suffering from chronic pancreatitis. Statistical analysis was performed on different questions.

**Results:**

Data on 229 patients (74% male and 26% female) were uploaded from 14 centres. Daily alcohol consumption was present in the aetiology of 56% of the patients. 66% of the patients were previously treated for acute exacerbation. One third of the patients had had previous endoscopic or surgical interventions. Pain was present in 69% of the cases, endocrine insufficiency in 33%, diarrhoea in 13% and weight loss in 39%. Diagnosis was confirmed with US (80%), CT scan (52%), MRI-MRCP (6%), ERCP (39%), and EUS (7,4%). A functional test was carried out in 5% of the patients. In 31% of the cases, an endoscopic intervention was performed with the need for re-intervention in 5%. Further elective surgical intervention was necessitated in 44% of endoscopies. 20% of the registered patients were primarily treated with surgery. The biliary complication rate for surgery was significantly smaller (2%) than endoscopy (27%); however, pancreatic complications were higher in the patients treated with surgery. Patients who smoked regularly needed significantly more surgical intervention following endoscopy (66.7% vs. 26.9%, p = 0.002) than non-smokers, and the ratio of surgical intervention alone was also significantly higher (27.3% vs. 10.8%, p = 0.004). The ratio of surgery in patients who smoked and drank was significantly higher (30.09% vs. 12.5%, p = 0.012) than in abstinent and non-smoking patients, similarly to the need for further surgical intervention after endoscopic treatment (71.43% vs. 27.78%, p = 0.004).

**Conclusions:**

According to the data analysed, the epidemiological data and the aetiological factors in our cohort differ little from European trends. The study highlighted the overuse of ERCP as a diagnostic modality and the low ratio of use of endoscopic ultrasonography. The results proved that alcohol consumption and smoking represent risk factors for the increased need for surgical intervention. Chronic pancreatitis should be treated by multidisciplinary consensus grounded in evidence-based medicine.

## Introduction

Chronic pancreatitis (CP) is a progressive inflammatory disease associated with structural and functional damage to the pancreas, causing pain, maldigestion and weight loss and thus worsening the quality of life. The clinical presentation is variable depending on the stage of the disease. The early stage disease (stage A) is dominated by recurrent clinical acute pancreatitis. In stage B, constant pain occurs with local complications from jaundice to pancreatic fistula, but exocrine and endocrine function is preserved. In end stage CP (stage C), pancreatic fibrosis leads to exocrine and/or endocrine function loss [1].

Although the pathomechanism of chronic pancreatitis is still poorly understood and evaluating a genetic predisposition and the effect of toxic agents (smoking and alcohol consumption) may open up potential for future research, the clinical features in the diagnosis and therapy ensure a great deal of evidence for lifelong management of the disease. Patients with chronic pancreatitis develop the clinical triad of abdominal pain and exocrine and endocrine pancreatic insufficiency. Despite the progressive fibrosis of the pancreatic tissue, the typical symptoms are not usually present, only in burn-out CP (after the loss of 90% of functioning pancreatic tissue) [2]. Frequently, only recurrent acute episodes show any evidence of the disease.

Though the diagnosis of CP can be obvious in advanced stage, confirming the disease in early stages without classical clinical symptoms is more challenging. Chronic pancreatitis is usually diagnosed with a combination of clinical presentation, imaging and pancreatic function tests. Due to the heterogeneous nature of the disease, a gold standard, universal treatment does not exist. The main goals of medical management of chronic pancreatitis are management of pain, exocrine and endocrine insufficiency, and treatment of possible complications, such as bleeding, biliary obstruction, pseudocyst formation or malignancy, requiring a personalized approach based on multidisciplinary decision-making.

Although numerous data are available on the clinical management of patients with CP, there is a lack of randomized controlled trials that provide strong evidence for individual diagnosis and treatment. There are only limited numbers of prospective cohorts available on the management of CP from Central Europe and no data from Hungary. The aim of our study was to collect data on patients suffering from chronic pancreatitis in a prospective manner and to find correlations from a multicentre database representing the epidemiological traits, diagnosis and treatment of the disease in Hungary.

## Patients and methods

The study was approved by the Scientific and Research Ethics Committee of the Medical Research Council (22254-1/2012/EKU).

The Hungarian Pancreatic Study Group (HPSG) collected data on patients suffering from CP in a prospective and voluntary manner from 2012 to 2014 and enrolled 229 patients from 14 Hungarian centres. Patients were enrolled in an official healthcare centre by a clinician. The study was open to all centres which managed to provide valuable and precise data after signing a Letter of Intent to Join. The patient was informed of the purpose of the research and blood sampling, and the Patient Informed Consent Form was signed.

Researchers actively contributing to the Biobank and Registry or collaborating researchers can access samples and clinical data. Samples and data are available free of charge and should be used for research purposes only. An application should be made for access. Data are available for others to analyse upon request only.

Demographic data (including age and gender), possible risk factors (frequency and total amount of alcohol consumption, smoking, body mass index (BMI), history of previous pancreatic disease and diabetes mellitus), aetiology, symptoms and clinical signs (such as fever, pain, diarrhoea, jaundice and weight loss), laboratory parameters, imaging techniques, conservative and interventional therapy (such as endoscopy and surgery) and complications were collected and assessed. All data were collected after patients gave their informed consent. Data was collected using the web-based electronic data collection method at the National Pancreas Registry (OPR).

Diagnosis of the disease was determined by the uploader under thorough supervision of the HPSG based on the M-ANNHEIM classification. Patients with dubious diagnosis were excluded. Diagnosis was based on imaging tests including abdominal ultrasound, computed tomography, magnetic resonance imaging (MRI), endoscopic retrograde cholangiopancreatography (ERCP) and endoscopic ultrasonography (EUS), including morphological findings typical of the different modalities. Relevant laboratory parameters were also collected. A cytological or histological diagnosis was performed using brush cytology during ERCP, fine needle aspiration biopsy (FNAB) or surgical biopsy/resection. The results of the pancreatic functional test were collected to prove the pancreatic exocrine insufficiency related to chronic pancreatitis.

The database included information on conservative and interventional treatment of CP. Data on enzyme substitution and anti-diabetic therapy were registered. The details of endoscopic treatment, such as stent type, number of interventions needed, complications and need for further intervention, were recorded. Similarly, we determined the type of surgical operation and complications.

In order to be able to process data and reach relevant conclusions, we supplemented insufficient data load by using the term Quality of Data (QoD), a ratio of the adequate data number of a specific question and the number of patients involved in the cohort.

Authorship policy: Authorship was given to contributors who uploaded data to the registry on at least five patients and were involved in acquisition, analysis and interpretation of data. Contributors who provided data on fewer than five patients are mentioned in the acknowledgements.

## Statistical analysis

For descriptive statistics, we calculated the case number, mean, standard deviation (SD), and minimum, median and maximum values in the case of continuous variables and the case number and percentage for categorical values. All statistical analyses were carried out with IBM SPSS Statistics v 20.0 (IBM Corporate, New York, USA). To compare the proportion between the subgroups, we used Pearson’s Chi-squared test or Fisher’s exact test. A p-value under 0.05 was considered statistically significant. Where the p-value was less than 0.1 but higher than 0.05, we suggested it as only a tendency.

## Results

Two hundred and twenty-nine patients were enrolled in the study (Table 1). The mean age of the population was 54.63 years. There were more males than females (73.8% vs. 26.2%, respectively).

**Table 1:** Patients’ epidemiological and anamnestic data.

		Number of patients (n)	Percentage (%)
***Gender***	*Male*	169	73,8
	*Female*	60	26,2
***Alcohol consumption (QoD*: *100%)***	*Never*	85	37,3
	*Occasionally*	40	17,5
	*Regularly*	103	45,2
***Smoking (QoD*: *99%)***	*>20 cigarettes/day*	56	24,7
	*10–20 cigarettes/day*	72	31,9
	*<10 cigarettes/day*	15	6,6
	*occasionally*	4	1,8
	*never*	79	35
***Previous hospitalisation due to acut pancreatitis (QoD*: *89%)***		151	66
***Previous endoscopic intervention (QoD*: *100%)***	***total***	72	31,4
	*ERCP-EST*	39	54,2
	*endobiliary stent*	27	37,5
	*Pancreatic duct stent stent*	0	0
	*pseudocyst drainage*	6	8,3
***Previous surgical intervention (QoD*: *100%)***	***total***	72	31,4
	*decompression*	8	11,1
	*drainage*	13	18,6
	*resection*	20	27,7
	*bilio-digestive anastomosis*	21	29,1
	*other*	10	13,9

### Risk factors and aetiology (QoD: 99%)

One hundred and forty-three patients (62.4%) were recorded as smoking regularly. Fifty-six patients (24.4%) smoked more than 20 cigarettes per day. Alcohol consumption was reported in 143 patients (62.4%), whereas 103 patients (56.7%) drank alcohol daily. Family history of CP was found in seven cases (3%). Genetic testing was only carried out in eight patients, of whom three proved positive (1.3%). Autoimmune origin was confirmed in one patient (0.4%). Recurrent acute pancreatitis was present in 75patients (32,75%).

### Symptoms and signs

The most frequent symptoms at the time of diagnosis in the period under examination was abdominal pain, which was present in 68.6% of all patients (QoD: 95%). Jaundice and fever were found in 11.5% and 11.35%, respectively (QoD: 100%).

### Exocrine insufficiency (QoD: 100%)

35% of the patients reported 3.03 kg/month average weight loss. Diarrhoea was recorded in 12.66% of the patients.

### Endocrine insufficiency (QoD: 100%)

Diabetes as an indicator of endocrine insufficiency was found on admission in 88 patients (38%) of the population under examination, of whom 37.5% were treated with insulin.

### Anamnesis

The reason for previous hospital admissions was acute exacerbations in 66% of the cases (QoD: 89%). 72 (31%) patients underwent an endoscopic intervention (we performed ERCP and EST in 54.2% of them, endobiliary stent implantation in 37.5% and endoscopic pseudocyst drainage in 8.3%). Previously, 72 (31.4%) patients had a different kind of surgical intervention (of these, 11.1% underwent decompression, 18.6% were treated with surgical pseudocyst drainage, 27.7% had pancreatic resection and 29.1% underwent bilio-digestive anastomosis) (QoD: 100%).

### Imaging (QoD: 100%)

As regards imaging examinations on admission, we performed abdominal ultrasonography in 184 patients (80%), CT scans in 120 (52%), MRI-MRCP in 14 (6.1%), diagnostic ERCP in 90 (39%) and endoscopic ultrasonography in 17 (7.4%). At least one US, CT or MRI was performed in 219 patients. Abnormalities characteristic of chronic pancreatitis based on the Cambridge criteria were found in 188 of these patients (86%). Similarly, in 95 patients who had MRCP or ERCP, 79 displayed typical abnormalities (83%). In 17 cases, EUS was performed, with only two cases showing normal gland structure (Table 2).

**Table 2:** Imaging modalities in the diagnostics of chronic pancreatitis.

Modality (QoD: 100%)	Number of patients (n)	Percentage (%)
***Ultrasonography***	184	80
***CT Scan***	120	52
***MRI-MRCP***	14	6,1
***ERCP***	90	39
***EUS***	17	7,4

### Functional tests (QoD: 81%)

As regards functional tests, only (13) C-triglyceride breath tests were performed in 5.2% of the patients with 3.5% positivity in the time interval under investigation.

### Conservative therapy (QoD: 98%)

131 patients (57%) received enzyme substitution. 71% of the diabetic patients were treated with insulin, 25% with oral antidiabetic drugs and 4% with both. 28 patients (12.5%) required pain killers after discharge.

### Endoscopic intervention

Endoscopic treatment was performed in 74 patients (32%) on admission. Endobiliary stents were implanted in 52% of all endoscopic interventions: of those, a single plastic stent was implanted in 50%, multiple plastic stents in 42.1%, a metal stent in 5.3% and a covered metal stent in 2.6% (QoD: 97%). 36.1% of the patients underwent ERCP-EST, 8.3% were treated with main pancreatic duct stenting, 1.3% received both pancreatic duct and biliary stents, and 4.1% had endoscopic pseudocyst drainage. In the endoscopically treated group, endoscopic re-intervention was required in 23% of the cases (QoD: 58%), while the ratio of further surgical intervention was 44% (QoD: 86%) (Tables 3 and 4).

**Table 3:** Endoscopic treatment.

Type of intervention (QoD: 97%)		Number of patients (n)	Percentage (%)
***ERCP-EST***		26	36,1
***Endobiliary stent***	***total***	38	52
	*Single plastic stent*	*19*	*50*
	*Multiple plastic stent*	*16*	*42,1*
	*Metal stent*	*2*	*5,3*
	*Covered metal stent*	*1*	*2,6*
***Wirsung duct stent***		6	8,3
***Wirsung and endobiliary stent***		1	1,3
***Pseudocyst drainage***		3	4,1

**Table 4:** Endoscopic compliations.

Type of complication (ENDOSCOPY)		Number of patients (n)	Percentage (%)
***Early complications (QoD*: *77%)***	*bleeding*	4	7
***Re-intervention needed (QoD*: *100%)***	***total***	12	31,6
	*stent removal*	5	35,7
	*stent exchange*	3	21,4
***Late complications***			
***Pancreatic (QoD*: *99%)***	*pseudocyst formation*	10	15,2
	*pancreatic fistule formation*	2	3
	*acut exacerbation*	1	1,5
	*necrosis/abscess*	1	1,5
	*pancreatic duct obstruction*	5	7,6
	*no complications*	47	71,2
***Biliary (QoD*: *100%)***	*biliary obstruction*	17	23
	*cholangitis*	3	4
	*no complications*	54	73

### Surgery

Surgery was performed in 49 patients (22%) from among the population under investigation in the period under examination (QoD: 100%). Pancreatic decompression was administered in 23.4% of the cases, while surgical drainage was done in 8% in cases where endoscopic drainage was not feasible or in one or two failed endoscopic attempts. The ratio of pancreatic organ-sparing resection was 32%. Bilio-digestive anastomosis was carried out in 26% of the patients. Reoperation was required in the postoperative period in one patient (2.4%) (QoD: 84%). The overall early complication rate of surgical interventions was 1.7% (Tables 5 and 6).

**Table 5:** Surgical treatment.

Type of surgery (QoD: 100%)	Number of patients (n)	Percentage (%)
*Pancreatic decompression*	11	23,4
*Surgical drainage*	4	8
*Organ sparing resection*	17	32
*Bilio digestive anastomosis*	12	26
*Other*	5	10,6

**Table 6:** Surgical complications.

Type of complication (SURGERY)		Number of patients (n)	Percentage (%)
***Early complications (QoD*: *22%)***		4	8
***Late complications***			
***Pancreatic (QoD*: *84%)***	*pseudocyst formation*	9	22
	*acut exacerbation*	4	9,7
	*necrosis/abscess*	4	9,7
	*pancreatic duct obstruction*	2	4,9
	*no complication*	22	53,7
***Biliary (QoD*: *100%)***	*biliary obstruction*	2	4
	*no complication*	47	96

### Early and late complications of endoscopic treatment

In the period under examination, 7% of the patients experienced bleeding, but 93% had no early complications (QoD: 77%). Due to stent occlusion, stents were removed in 35.7% of the cases and exchanged in 21.4% of them. The ratio of patients who required later stent implantation was 28% (QoD: 100%).

In the majority of patients (47 patients; 64%), there were no pancreatic complications. The most frequent late pancreatic complication was pseudocyst formation in 15.2% of the cases, followed by obstruction of the duct of Wirsung in 7.6%, fistula formation in 3%, and abscess and recurrent acute exacerbations in 1.5% each (QoD: 89%). Biliary obstruction was observed in 17 cases (23%) and cholangitis in three (4%), while 73% of the patients had no late biliary complications (QoD: 100%). As regards distant organ complications, sepsis occurred in 4% of the patients, respiratory distress in 2% and multi-organ failure (MOF) in 1% (QoD: 99%).

### Early and late complications of surgical treatment

Of the 49 operations performed, only one anastomosis leakage was recorded; other non-detailed early complications occurred in three cases on according to admission records.

Pancreatic pseudocyst formation was also the most frequent late complication in nine patients (22%), followed by acute exacerbations and necrosis/abscess formation in four each (9.7% each) and main pancreatic duct obstruction in two (4.9%). In 53.7% of the population under investigation that underwent surgical intervention, there were no late complications (QoD: 84%). Biliary obstruction was observed in two patients (4%), while there were no late biliary complications in 96% of them (QoD: 100%). Sepsis occurred in one patient (2%), while MOF and respiratory complications were noted in two each (4% each) (QoD: 100%).

### Correlations between alcohol consumption and the course of the disease

In the population under examination, patients who consumed alcohol regularly were more likely to have a previous acute exacerbation (76.3% vs. 67.5%) compared to abstinent patients, but the difference was not significant (p = 0.174). The incidence of exocrine insufficiency (58.99% vs. 58.33%, p = 0.923) and diabetes mellitus (38.46% vs. 38.82%, p = 0.821) did not differ significantly in the groups under examination; however, blood sugar level measured on admission was slightly higher in patients who consumed alcohol regularly (8.61 vs. 7.39mmol/l, p = 0.383, QoD: 62–67%). BMI showed almost the same value in the groups under investigation (22.11 vs. 21.69, p = 0.622, QoD: 69–78%).

In the period under examination, there was not significantly more endoscopic intervention required in patients who drank alcohol (32.9% vs. 32.1%), but, surprisingly, further surgical intervention was needed after endoscopy in more of those patients than among abstinent patients (57.9% vs. 36%, p = 0.089). There was no significant difference (p = 0.636, QoD: 70–85%) in the number of early complications, such as bleeding (3 vs. 1%) or perforation (0 vs. 0%), associated with endoscopy. The number of re-interventions needed during hospitalization was also statistically identical.

Patients who drink alcohol underwent surgery more often (23% vs. 9.8%, p = 0.233). There was no difference in the rate of early complications.

The late complications related to endoscopy (pancreatogenic: 26.83% vs. 36%, p = 0.432, QoD: 87–93%); biliary: 23.4 vs. 29.63%, p = 0.792; sepsis-MOF: 8.7 vs. 7.41%, p = 1) showed no difference with regard to regular alcohol consumption. After surgery, biliary obstruction (3.13 vs. 7.145%, p = 0.521) and severe complications resulting in injury to other organs (6.25 vs. 21.43%, p = 0.157) were similar with no significant difference. Surprisingly, pancreatogenic complications (recurrent acute exacerbations, fistula formation, pancreatic duct obstruction, pseudocyst formation, necrosis/abscess) proved to be higher in abstinent patients compared with patients who consumed alcohol.

**Fig 1:**
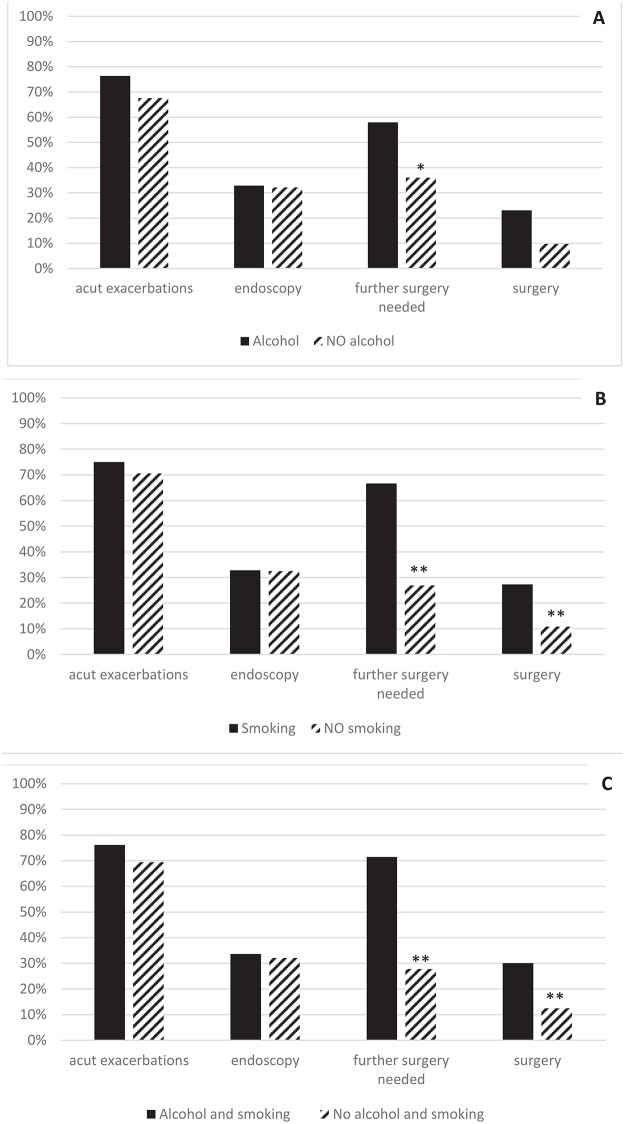
**Correlations between alcohol consumption (A), smoking (B), smoking with alcohol consumption (C) and the course of the disease** prevalence of acute exacerbation; hospitalisation due to endoscopic intervention; need for futher surgery after endoscopic intervention; hospitalisation due to surgery.

Dividing the alcohol-consuming patients into three subgroups– 1–9U/day, 10–19U/day and >19U/day–according to the amount of alcohol consumed, we examined any correlation between the dose of alcohol and the course of the disease (QoD: 35.66%). Hospitalization due to acute exacerbations correlated with the dose of alcohol consumption (63.16 vs. 76.19 vs. 87.5%, p = 0.388). Because of the low number of available data, it was not possible to confirm any further correlation (Fig 1A).

### Correlations between smoking and the course of the disease

Patients who smoked regularly were more likely to have a previous acute exacerbation (75% vs. 70.5%) in their anamnesis compared to non-smoking patients with no significant difference (p = 0.48). Incidence of exocrine insufficiency (58.99% vs. 55.42%, p = 0.56) and diabetes mellitus (36.36% vs. 43.37%, p = 0.566) did not differ significantly in the groups under examination, while blood sugar level measured on admission was slightly higher in smoking patients (8.37 vs. 7.39mmol/l, p = 0.664, QoD: 65–69%). BMI showed a non-significant difference in the groups under examination (21.57 vs. 22.52, p = 0.304, QoD: 71–72%).

In the period under examination, smoking patients did not need more endoscopic intervention (32.9% vs. 32.5%), but significantly more further surgical intervention (66.7% vs. 26.9%, p = 0.002) was required following endoscopic treatment than in the case of non-smokers.

During the study period, significantly more surgical interventions were observed in smoking patients (27.3% vs. 10.8%, p = 0.004) than in non-smokers.

There was no significant difference in early and late complications after endoscopy or surgery in the groups under examination (Fig 1B).

### Correlations between smoking, alcohol consumption and the course of the disease

In the population under investigation, the patients who smoked and consumed alcohol regularly were more likely to have had previous acute exacerbations (76.19 vs. 67.31%, p = 0.237)) than non-smoking and abstinent patients. Incidence of enzyme substitution therapy (57.8 vs. 53.57%, p = 0.604) did not differ significantly in the groups under examination, while blood sugar level measured on admission was slightly higher in smoking and alcohol-consuming patients (8.78 vs. 7.52mmol/l, p = 0.522, QoD: 62–63%). However, significantly more patients suffered from diabetes mellitus (treated with insulin: 22.12 vs. 10.71%, p<0.001; treated with OAD: 64.6 vs. 26.79%, p<0.001) in the smoking and alcohol-consuming group compared to the abstinent group.

BMI showed a non-significant difference in the groups under investigation (21.71 vs. 22.01, p = 0.792, QoD: 77–78%). In the period under examination, similar ratios of smoking and alcohol-consuming patients needed endoscopic intervention (33.63 vs. 32.14%, p = 0.847) compared to non-smoking and abstinent patients; however, significantly more patients required further surgical intervention after endoscopy (71.43 vs. 27.78%, p = 0.004). There was no significant difference in the number of early complications, such as bleeding or perforation associated with endoscopy, and the number of re-interventions needed during hospitalization. Similarly, no significant difference was observed in late complications.

Smoking and alcohol-consuming patients had surgery significantly more often (30.09 vs. 12.5%, p = 0.012) than non-smoking and non-drinking patients (Fig 1C).

### Correlations between endocrine and exocrine insufficiency and the course of the disease

We examined whether diabetes mellitus and known exocrine insufficiency treated with enzyme substitution resulted in a significant influence on the course of the disease. We could not prove any tendency or significant difference between the groups under examination either related to enzyme substitution or to diabetes mellitus. The rate of acute exacerbations (DM: p = 0.247; enzyme substitution: p = 0.439), endoscopic interventions (DM: p = 0.106; enzyme substitution: p = 0.97), surgery (DM: p = 0.721; enzyme substitution: p = 0.481), and complications proved to be identical without any tendency.

## Discussion

There are very limited data available on the aetiology, symptoms, management and outcome of CP in Hungary. This cohort in CP is the first attempt to collect generally valid data on the epidemiology, diagnosis and treatment of the disease, using data from patient uploads based on enthusiasm but not obligation on the part of Hungarian pancreatologists. However, epidemiological data may be collected from a single record for the patient. Due to the chronic behaviour of the disease, relevant information on the appropriate treatment, possible complications and course of the disease can be obtained from substantial records of repeated meetings with the patient. This thorough patient follow-up needs to be developed in the future. In this cross-sectional study, we presented the initial data on the first Hungarian cohort in chronic pancreatitis. Without knowing the epidemiological data in Hungary and ensuring a comprehensive patient enrolment, we would be hard-pressed to assert that these data represent the Hungarian population well. However, after the national centres are linked, the database will cover the population with increasing precision. In order to be able to process the data thoroughly, we used the term quality of data (QoD) described above. Nevertheless, with the known limitations, the study provides a good reflection of the course of the disease and the difficulties of diagnosis and treatment in Hungary.

According to various studies, the incidence of CP varies from 4/100,000 in the US [3] to 13.4/100,000 in Finland [4]. The incidence is 11.9/100,000 in Japan [5], 10/100,000 in Denmark [6], 6.4/100,000 in Germany [7], 7.7/100,000 in France [8] and 7.8/100,000 in the Czech Republic [9]. The limits of epidemiological surveys derive from the need for long-term follow-up and the variability of the severity of the disease [10]. The median survival time in alcoholic CP is 20–24 years after the onset of the disease [11]. Unfortunately, there is no current data on the incidence and median survival time for CP in Hungary.

There is evidence of a correlation between CP and pancreatic cancer (PC). Besides the fact that smoking and drinking could be an independent risk factor for pancreatic cancer, it has been demonstrated that in the case of clinically proven CP, the risk for PC is higher than in the average population [12]. According to HPSG multicentre data collection and analysis of pancreatic tumours (PC), the ratio of the presence of CP in the case of PC was 3.7% [13]. As discussed previously, the cross-sectional CP cohort does not contain data on follow-ups. Further development of the registry and ongoing prospective data collection may allow us to obtain valuable information on that topic. Although the cause of CP is regarded as a multifactorial disease [14], the most significant cause of chronic pancreatitis in adult patients is alcohol consumption, except in South India and China, where idiopathic pancreatitis was the most common cause [15]. A multicentre study from Italy showed that 34% of CP cases were caused by excessive alcohol consumption [16]. That figure was 65.4% in the Czech Republic [9], 44% in the US, 95% in Australia and 54% in Japan [15]. In the Hungarian cohort, total alcohol-related CP was 62% (45% of all CP cases consumed alcohol regularly and 18% admitted to occasional alcohol use). This number roughly correlates with other national studies, but it is important to note that some patients may hide their alcohol consumption habit because of its social effects.

Smoking was proved to be another risk factor for CP [17–20], and it seems to reinforce the toxic effect of alcohol in the pancreas [19, 21]. However, other studies suggest that cigarette smoking alone is a risk factor [22]. In our study, 63% of patients still smoked regularly (25% smoked more than 20 cigarettes/day, 32% 10–20 cigarettes/day, and 6% fewer than 10 cigarettes/day), which is a higher percentage than in the normal population in Hungary (32.3% of males and 23.5% of females were smokers in Hungary in 2012).

The role of smoking and alcohol consumption as risk factors for developing CP is well-examined, although the effect on the course of the disease is still unclear. Studies have shown that continuous smoking increases the risk of pancreatic calcification [23] in alcohol-related [19, 24] and idiopathic chronic pancreatitis [20, 25]. A Spanish study from 2014 found an association between tobacco usage and pancreatic exocrine insufficiency in CP [23]. In our study, we established no significant difference between smokers and non-smokers in the need for pancreatic enzyme substitution therapy (59% and 55.4%). Studies found no association between alcohol consumption and pancreatic calcification [23, 24], but demonstrated that cessation of alcohol reduces pain [26]. Abstinence decreased the number of acute exacerbations in CP [27]. In our study, patients who consumed alcohol regularly were more likely to have experienced previous acute exacerbation compared to abstinent patients. Our study showed that alcohol and tobacco usage alone increased the number of acute exacerbations and the need for endoscopic interventions and surgery and that more patients were referred for further surgical interventions after endoscopic treatment. Combined alcohol consumption and smoking significantly increased the risk of the need for more invasive therapy, suggesting the reinforcing effect of these exogenic factors. Diabetes mellitus is also more frequently present in these patients. In line with our results, Pan et al. showed that the risk of developing DM in patients with CP is influenced by modifiable factors, including alcohol abuse and distal pancreatectomy. It would be interesting to find correlations between the dose of alcohol and the course of the disease. Regrettably, due to the low amount of available data, we could not detect any statistically meaningful correlations. With further improvement to our registry, more thorough data collection is anticipated. However, to identify patients with familial CP–similarly to autoimmune pancreatitis—and find correlations with the other etiologies would be fruitful in term of early diagnosis of these patients, our cross-sectional cohort does not contain enough valid data for that analysis. The improved prospective data collection will open the door for more detailed investigations.

Pain is the hallmark symptom of CP, usually epigastric radiation to the back or to the left upper abdomen, and it is the most common cause of clinical admission [28]. In our cohort, 68% of the patients suffered from pain; in other studies, this varies between 80% and 96% [29–32].

In stage B of chronic pancreatitis, development of common bile duct stricture (CBS) is expected in 3–46% of cases [33–40]; in our cohort, 11% of the patients suffered from jaundice.

Exocrine insufficiency characterized by steatorrhea and loss of weight occurs in end-stage pancreatitis in approximately 30% of cases [41, 42]. 35% of the patients in our study reported significant weight loss, and diarrhoea was recorded in 12.66% of the cases. However, a distressingly low portion of patients were examined with a functional test (5.2%). Endocrine insufficiency was observed in 33% of our cohort, while another study showed 50–75% [43]. However, this is probably a lower percentage than the actual numbers [44].

Diagnosing CP can be challenging, especially in the early stages, because sometimes no radiomorphological signs or laboratory abnormalities can be found. In our cohort, the typical abnormalities were found in 83–86% of the patients, depending on the imaging modality. There is a broad spectrum of imaging techniques that can be used. Transabdominal ultrasound (US) is a relatively cheap, easily accessible, non-invasive and rapid diagnostic tool. It can be employed to detect pancreatic calcifications, pseudocysts and complications of CP, such as common bile duct obstruction and splenic or mesenteric vein obstruction. Unfortunately, bowel gas and body composition can make the process difficult, and there is no correlation between pancreatic exocrine function and the number of calcifications [45]. The sensitivity of transabdominal US is between 60 and 81%, while specificity is between 70 and 97% [46–48]. In our cohort, US was performed in 80% of the patients. Computed tomography is regarded as one of the best initial imaging tests. It is widely available and permits a detailed evaluation of pancreatic parenchyma, helping rule out pancreatic malignomas. CT is superior to US in detecting pseudocysts and complications of CP. The sensitivity and specificity of CT are 75–90% and 80%, respectively [49]. ERCP was long the gold standard for CP diagnosis and staging, with sensitivity of 70–95% and specificity of 90% or more, but with a significant morbidity (3 to ≥40%) and mortality (0.1–1%) [50]. It is therefore no longer employed as a diagnostic tool. MRCP is a non-invasive imaging test, which does not use ionizing radiation and provides an excellent image of the main pancreatic duct, with sensitivity of 88% and specificity of 98% [51]. Secretin-enhanced MRCP allows for a quantitative assessment of exocrine pancreatic function by measuring the duodenal filling [52] and provides a more accurate way to identify small-duct disease in mild chronic pancreatitis [53]. The Hungarian Pancreatic Study Group (HPSG) prepared an evidence-based guideline for CP, which does not recommend the use of the ERCP as a diagnostic tool because of its morbidity and mortality rates. Unfortunately, 39% of participating patients underwent diagnostic ERCP. Endoscopic ultrasound (EUS) is a very sensitive diagnostic tool, allowing for the evaluation of the pancreatic parenchyma and duct system with sensitivity and specificity of 80–100% [54], but it is observer-dependent and has a tendency toward over-diagnosis [55]. Unfortunately, EUS was used in only 7.4% of all patients in our cohort, a result explained by the still low number of imaging devices in the country.

However, the diagnosis of chronic pancreatitis is mainly based on imaging techniques, while functional tests can be helpful in inconclusive cases. The fact that neither indirect nor direct functional tests are widespread and available in Hungarian centres has resulted in the fact that only 5.2% of the entire population under examination had undergone any kind of functional testing. Despite poor functional testing for CP, enzyme substitution was administered in 57% of the patients based on clinical symptoms and radiomorphological changes. Steatorrhea only occurs when lipase secretion is ≤10% [56]. It is important to distinguish between pancreatic exocrine insufficiency and CP as up to 20% of CP cases with exocrine insufficiency presented with no history of pain [57]. In Hungarian clinical practice, enzyme substitution therapy is reimbursed in the presence of steatorrhea or maldigestion. In our study, 52% of the patients received enzyme substitution therapy.

The therapy for chronic pancreatitis is complex and based on lifestyle changes. In the case of alcoholic CP, complete elimination of alcohol reduces the pain [26] in 50% of patients [58], and there is increasing evidence that tobacco use plays an important role in CP [19]. In our cohort, we have no information on how many of our patients remained abstinent and/or stopped smoking and how this affected their quality of life. Pain is a major clinical problem in CP patients. Worldwide, pain management follows the WHO “pain relief ladder” recommendation; in our study, 12.5% of all the patients required continuous analgesia and 87.4% took painkillers episodically. Unfortunately, we have no information on the kinds of drugs they used.

Managing CP patients is a complex task; treatment requires different strategies depending on the stage, aetiology, morphological changes and various symptoms of the disease. Basically, the treatment requires a multidisciplinary consensus.

Endoscopic intervention may be feasible for the drainage of pancreatic pseudocysts and jaundice caused by CBS as well as for main pancreatic duct calcifications with proximal juxtapapillary stenosis. In pain management, endoscopic treatment showed good results for short-term symptomatic disease (≤4 years), if there was no pancreatic duct stricture and if obstructive calcifications were restricted to the pancreatic head [59]. Besides the latter, there are two groups of patients where primary endoscopic treatment seems to be a better choice than surgery; children with hereditary pancreatitis [60] and patients who have portal vein thrombosis and are unfit for surgery [30]. The indication for pseudocyst endoscopic drainage is a symptomatic disease (pain, abdominal discomfort, CBS and gastric outlet syndrome), 3–6 months’ wait-and-see after the diagnosis and a minimum size of 3cm [61]. Long-term clinical success may be 70–90% [62]. In our cohort, endoscopic pseudocyst drainage was done in 4.17% of the patients and two out of three patients had recurrent pseudocysts as a late complication.

In our study, endobiliary stent implantation was performed in 52% of the patients (50% of these interventions involved a single plastic stent, 42.1% multiple plastic stents, 5.3% a self-expandable metal stent and 2.6% a covered metal stent). An endoscopic stent implant of longer than 12 months–particularly in the case of calcification in the pancreatic head–is inferior to surgery. It only provides a short-term solution, the long-term success rate is poor and only one out of four strictures is treated effectively with this method [63]. Obstructive jaundice recurs in up to 88% of cases [62] and with the presence of pancreatic head calcification the risk of failure is 17-fold (95%) in a twelve-month period. During the median follow-up, 49.2% of the patients required surgical intervention after endoscopic stent implantation [64]. Bilio-digestive anastomosis during surgery provides a better result; only 18% of the patients developed stricture after the operation [65]. In our study, 23% of all patients needed further stent implantation (QoD: 58%). More importantly, a high number of patients (44%) required surgical intervention after endoscopic biliary drainage, indicating the priority of surgical procedures in chronic pancreatitis with biliary obstruction.

Pain, the hallmark symptom of CP, is the major indication for surgery, which is the most effective long-term form of pain therapy for chronic pancreatitis. Two randomized controlled studies provided significantly better pain management with surgical intervention involving a pancreatojejunostomy than with endoscopic treatment [66] [67]. 97% of patients with CP may suffer from abdominal pain, between 35% and 49% experience CBS, and between 6% and 12% have duodenal stenosis [68, 69]. In our cohort, pain occurred in 68% of the cases and CBS in 11%, but, unfortunately, we have no information on DS. The goals of surgical interventions besides pain relief are to preserve as much functional pancreas tissue as possible by correcting anatomical changes such as CBS and DS. In CP, the source of pain can stem from two sources: parenchymal compression due to the obstructed pancreatic duct system and the alteration of intrapancreatic nerve fibres and the activation state of intrapancreatic glia due to chronic inflamed pancreatic tissue, especially in the pancreatic head. The head is considered the pacemaker of pain, causing neuropathic pain and visceral neuropathy [70]. As we noted before, endoscopic pseudocyst drainage is recommended, unless the anatomical situation or the cyst content does not allow such an intervention. The aim of surgical drainage procedures is the drainage and decompression of pancreatic tissue due to the obstructed pancreatic duct system. The first method was described by Duvall in 1953: pancreatic tail resection and splenectomy with retrograde drainage of the main duct into a defunctioning jejunal loop. In 1958, the Puestow–Gillesby modification added a longitudinal opening of the pancreatic duct to the Duvall procedure and achieved wider drainage of the pancreatic duct system. The Partington–Rochelle procedure consists of a side-to-side lateral pancreaticojejunostomy, without a pancreatic tail and spleen resection, sparing pancreatic tissue and preventing endocrine and exocrine pancreatic insufficiency. The procedure relieves chronic abdominal pain in 66–91% of cases with a mean follow-up of 3.5–9.1 years; unfortunately, 30% of patients experience no pain relief due to the chronic inflamed pancreatic head [71]. In our study, 8% of the patients underwent a drainage procedure, but we do not know the pain recurrence rate in the absence of long-term follow-up. The inflamed, enlarged pancreatic head mass causes CDS and DS. Pancreaticoduodenectomy (PD) was the only solution until 1972, when Beger described the duodenum-preserving pancreatic head resection (DPPHR). In this procedure, the stomach, the duodenum and the extrahepatic bile ducts are spared. With the decompression of the intrapancreatic common bile duct and the resection of the inflamed pancreatic head, the common bile duct can be opened and sutured to the bottom of the resected cavity [72]. In 1987, Frey and Smith developed a hybrid DPPHR adding a LPJ. The Berne modification to the Beger procedure avoids the transection of the pancreas, instead performing a scoop-like pancreatic head resection with one pancreaticojejunal anastomosis. In the case of the Beger procedure, after a median follow-up of 5–7 years, less than 10% of patients experienced recurrent pain [73]. In contrast, 75% of patients were pain-free with the Frey procedure with a follow-up of 3–4 years, but 13% experienced no pain relief [74]. Compared to PD, DPPHR is equally effective in terms of postoperative pain relief, overall morbidity and incidence of postoperative endocrine insufficiency [75]. A metaanalysis from 2015 compared the Frey and Beger procedures, and pain relief was achieved in 89% of cases using the Frey procedure in PD with a shorter operation time and lower overall morbidity [76]. 18% of patients who had undergone DPPHR developed a stricture in the reinsertion site, while it was only 4% in PD [65]. If DS is present or the probability of malignancy occurs, standard PD is recommended. In our study, the rate of pancreatic resection was 32%, pancreatic decompression was 23.4%, surgical drainage was 8% and bilio-digestive anastomosis construction was 26% with an acceptable early complication rate and rate of required reoperation in the early postoperative period as well as a definitely smaller complication rate on the biliary tract, compared to endoscopic interventions.

## Conclusions

Chronic pancreatitis should be treated by multidisciplinary consensus using evidence-based medicine. Data should be revised continuously in accordance with the chronic nature of the disease. Conclusions from the first nationwide prospective data collection effort in Hungary provide important information to improve treatment of the disease and define the role of endoscopy and surgery. However, the quality of data collection requires further development with improvement to the registry. In our cohort, (1) the epidemiological data are comparable to international studies. (2) The aetiological factors differ little from European trends. (3) The number of times diagnostic ERCP is used should be reduced, while use of EUS should be increased. (4) Our results proved that alcohol consumption and smoking represent a risk factor for the increased need of surgical intervention, suggesting that the elevated number of patients cannot be treated with conservative and less invasive endoscopy. (5) The role of surgery in the treatment of chronic calcifying pancreatitis with biliary obstruction should be highlighted. Future plans by the Hungarian Pancreatic Study Group include improving the quality of data collection and expanding the database to other Central and Eastern European countries.

## Supporting information

S1 Table Data of alcohol consumption, smoking, smoking with alcohol consumption, dose of alcohol, enzyme substitution and diabetes mellitus. (XLSX)
